# The plastic cellular states of liver cells: Are EpCAM and Lgr5 fit for purpose?

**DOI:** 10.1002/hep.28469

**Published:** 2016-03-17

**Authors:** Meritxell Huch, Laurent Dollé

**Affiliations:** ^1^ Wellcome Trust/Cancer Research UK‐Gurdon Institutethe Wellcome Trust‐Medical Research Council Stem Cell Institute, and Physiology, Development, and Neuroscience, University of CambridgeCambridgeUK; ^2^ Laboratory of Liver Cell BiologyDepartment of Basic Biomedical SciencesFaculty of Medicine and PharmacyFree University BrusselsBrusselsBelgium

## Abstract

Adult liver cells have been considered restricted regarding their fate and lineage potential. That is, hepatocytes have been thought able only to generate hepatocytes and duct cells, only duct cells. While this may be the case for the majority of scenarios in a state of quiescence or homeostasis, evidence suggests that liver cells are capable of interconverting between cellular states of distinct phenotypic traits. This interconversion or plasticity had been suggested by classical studies using cellular markers, but recently lineage tracing approaches have proven that cells are highly plastic and retain an extraordinary ability to respond differently to normal tissue homeostasis, to tissue repair, or when challenged to expand *ex vivo* or to differentiate upon transplantation. Stemness, as “self‐renewal and multipotency,” seems not to be limited to a particular cell type but rather to a cellular state in which cells exhibit a high degree of plasticity and can move back and forth in different phenotypic states. For instance, upon damage cells can dedifferentiate to acquire stem cell potential that allows them to self‐renew, repopulate a damaged tissue, and then undergo differentiation. In this review, we will discuss the evidence on cellular plasticity in the liver, focusing our attention on two markers, epithelial cell adhesion molecule and leucine‐rich repeat‐containing G protein‐coupled receptor 5, which identify cells with stem cell potential. (Hepatology 2016;64:652‐662)

**Abbreviations**
EpCAM: epithelial cell adhesion molecule
Lgr5: leucine‐rich repeat‐containing G protein‐coupled receptor 5

## Stem Cell Fate and Stem Cell Potential: Different Sides of Cellular Plasticity

The stem cell state is defined by the ability of cells to fulfill the two following criteria: self‐renewal and multipotency.^1^ Several approaches have been used to identify cells that exhibit stem cell characteristics. *In vivo*, long‐term label‐retaining and genetic lineage tracing have been commonly used to identify both quiescent and actively cycling stem cells in several tissues.^1^ Alternatively, *in vitro* clonogenicity and multilineage differentiation as well as long‐term repopulation following transplantation have been regarded extensively as assays to demonstrate stem cell potential.^1^

Of note, stem cell fate and stem cell potential might have not always been adequately used. Stem cell fate indicates a cell that already fulfills the stem cell criteria, while stem cell potential represents a cell with the competence to acquire a stem cell state, depending on the environment or condition. Confusion might have been caused by the extensive plasticity of animal cells. Cellular plasticity is understood as the propensity of a cell to, under certain circumstances, acquire the biological properties of other cells.^2^ Because stem cell potential can be defined as the ability of cells (differentiated cells or progenitors) to acquire a stem cell state, stem cell potential would therefore be a specific manifestation of plasticity.^2^ On the other hand, one could also consider that this return to a more primitive state is a form of *in vivo* reprogramming. However, “reprograming” is associated with a complete reversion to a pluripotent state, as seen in Gurdon's tadpole experiments.^3^ In this review we use “plasticity” to mean the ability of cells to acquire other cellular fates, distinct from reprograming; and thus, acquisition of a tissue‐restricted stem cell fate or potential would be one form of plasticity.

Several authors have suggested the existence of plasticity in adult liver cells,^4^, ^5^, ^6^, ^7^ but advances in mouse genetic engineering, imaging tools, and the possibility of culturing cells *in vitro* have provided further evidence for cellular plasticity in the liver and other organs. Here, we review the evidence of liver cellular plasticity. We will use epithelial cell adhesion molecule (EpCAM) and leucine‐rich repeat‐containing G protein‐coupled receptor 5 (Lgr5) as examples of markers that identify cells with cellular plasticity and stem cell potential in the liver.

## Cellular Plasticity: An Old Player in the New Viewpoint of Looking at Liver Repair

Increasing evidence of stem cell behavior in the intestine, hair follicle, and bone marrow suggests that cells often exist in two distinct states: an active stem cell state and a potential state that appears upon stem cell ablation. Studies on both intestinal and hair follicle cells show that when the stem cell pool is ablated, those cells which retain stem cell potential (usually early descendants of the stem cell) acquire properties of a stem cell (potential/plasticity), such as the ability to repair tissue and reinstate homeostasis (nicely reviewed by Blanpain and Fuchs^2^). Similarly to the intestine or skin, organs with slow physiological turnover, such as the lung, also possess a high degree of cellular plasticity. For instance, after ablation of airway stem cells, lineage tracing demonstrated that luminal secretory cells had dedifferentiated into multipotent basal stem cells.^8^ This capacity of cells to acquire a stem cell state may have a more general role in the regeneration of many tissues, including the liver.

The primary functional unit of the liver is the hepatic lobule or acinus, a structure resulting from the interaction between epithelial (hepatocytes and ductal cells), endothelial (sinusoidal cells), and mesenchymal (portal fibroblasts and stellate cells) cells.^9^

In the liver, during embryonic development, hepatoblasts behave like stem cells as they are capable of self‐duplicating while giving rise to hepatocytes and ductal cells (elegantly reviewed by Miyajima et al.^9^). During adulthood, cellular turnover is rather slow, with a period of more than several months.^10^ Extensive lineage tracing approaches in the mouse model indicate that if adult liver stem cells exist, their contribution to normal homeostasis is negligible, at least in the mouse model, with the exception of one report that demonstrated, using genetic lineage tracing based on Sox9CeER, that adult hepatocytes can also derive from specialized ductal progenitors.^11^ However, other studies did not find evidence for such liver progenitors.^12^, ^13^ Also, recently, a subset of centrilobular hepatocytes has been shown to contribute to normal homeostasis of the hepatocyte compartment.^14^, ^15^ On the other hand, using clonogenic assays, it has been reported that EpCAM^+^ human liver cells, isolated from healthy fetal, neonatal, pediatric, and adult^16^, ^17^ donors, display characteristics of liver stem/progenitors both *in vitro* and *in vivo* after transplantation. The latter could be understood as the ability of some resident cells to harbor stem cell potential during homeostasis. However, it is worth noting that clonogenic assays isolate the cells from their environment, which could trigger activation of a stem cell state as a result of damage to the tissue, as happens during a regenerative response. Therefore, as we will discuss below, the results from clonogenic assays could also be interpreted as the responses of cells to the external cue of being isolated from their tissue, which does not necessarily reflect what happens during *in vivo* homeostasis.

**Figure 1:**
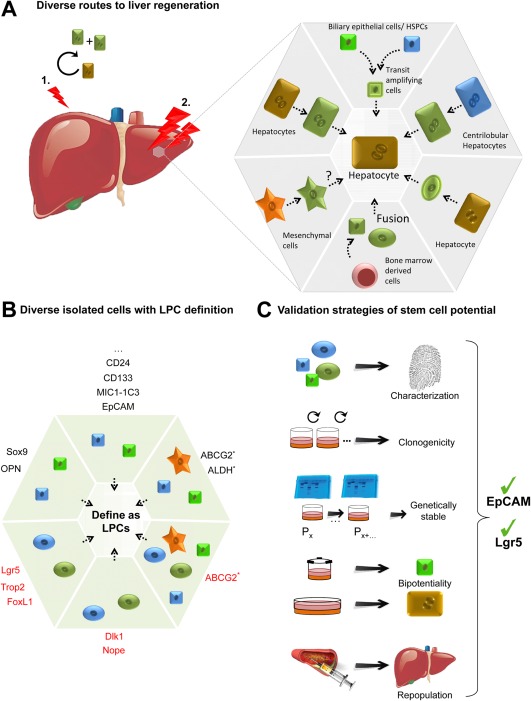
Plasticity concept. (A) Diverse routes lead to liver regeneration. While in a quiescent, homeostatic state (1), tissue is maintained primarily by proliferation of the subpopulations of mature hepatic cells capable of cell division; upon damage (2) various cell sources have been described to be involved in the process of hepatic repair. Each of them is illustrated in one‐quarter of the gray hexagon; at the outer limits, the various cell sources are represented at their initial state, while the center of the hexagon represents their ultimate goal: to produce new hepatocytes (but also new duct cells or ducts). While each cell source has its color, their differentiated state is illustrated in green. Stromal cells are shaped as stars; bone marrow‐derived cells are represented as round cells; particular hepatocytes from the central vein are highlighted in blue. Biliary cells (and hepatic stem/progenitor cells) and transit‐amplifying cells are represented as small green and blue cells, respectively. (B) Diverse isolated cells are defined as liver progenitor cells. Using flow cytometry‐based cell separation methods in combination with cell surface markers or functions or genetic tracers, liver cells with stem cell potential have been isolated as viable cells. Black illustrates cell surface and genetic markers used in healthy livers, while red indicates markers used upon damage. Asterisk indicates function. Arrows indicate that whatever the nature of the isolated cells is, they all converge to the definition of “liver progenitor cells.” (C) Validation strategies of stem cell potential. Isolated cells are subjected to *in vitro* culture to evaluate their bidirectional differentiation, clonogenic potentials, and organoid formation and *in vivo* to repopulate the liver upon transplantation. Lately, genetic stability studies have been introduced. Until today, only EpCAM and Lgr5 have completed successfully this list. Abbreviations: HSPC, hepatic stem/progenitor cell; LPC, liver progenitor cell.

The liver excels in its extensive damage‐repair response (see Fig. 1A).^18^ The cells responsible for the facultative regenerative response of the liver have been the subject of extensive investigations. This has led to two schools of thought: the followers and the opponents of the existence/activation of a progenitor response that would contribute to the repair of the tissue after damage. On the one hand, mouse lineage‐tracing approaches in combination with specific cell markers have allowed the identification of cells that upon damage will differentiate into hepatocytes and/or ductal cells.^11^, ^19^, ^20^, ^21^, ^22^ However, in all of these studies, the lack of *in vivo* clonal analysis hampers the ability to conclude whether these cells are truly bipotential. Thus, until this is fully addressed, the existence of true bipotential cells induced after damage remains undetermined. Also, *in vitro* studies from several groups indicate that isolated progenitors from mouse injured livers display bipotentiality *in vitro* and *in vivo* following transplantation into FAH mutant mice.^19^, ^20^, ^22^, ^23^ Also, recently, Kaneko et al. showed that upon damage biliary cells expand toward the injured area, suggesting that the expanded biliary branches could contribute as a source or as a niche during the regeneration response.^24^ On the other hand, reports using virus‐mediated Cre lineage‐tracing approaches have recently ruled out the existence of progenitor‐driven regeneration in the mouse.^25^, ^26^ One explanation for this paradox could be that liver pathologies in these models are not sufficiently severe, so remaining “healthy” hepatocytes can still extensively proliferate and repair the lost tissue. Notably, in zebrafish, genetic ablation of the hepatocyte compartment followed by lineage tracing resulted in ductal cells dedifferentiating and acquiring a stem cell fate, where biliary tree stem/progenitors repair the damaged liver.^27^ Also, upon complete senescence of the hepatocyte compartment, Lu and colleagues observed a similar widespread ductular reaction in the mouse.^28^ Moreover, recent studies indicate that following transplantation and injury, mouse hepatocytes can acquire a ductal phenotype and stem cell state and can differentiate toward hepatocytes and ductal cells upon demand.^29^ Similarly, in human liver failure, ductal cells are detected close to clusters of hepatocytes that also express ductal markers.^30^ Whether in humans the ductal cells derive from hepatocytes or the inverse is true might be difficult to determine without the possibility of tracking the cells *in vivo*.

Overall, these studies suggest that the adult liver cellular state (either hepatocyte or duct) is not fixed but can be modulated upon request. Differentiated states can be dedifferentiated or pushed to a more “stem cell state” upon demand. In these reports, adult liver cells fulfill the stem cell criteria, whereby they will proliferate and differentiate depending on the type and extent of the damage and the model organism studied. The differences that are being observed might be due to the type of injury, the type of model (human, mouse, rat, zebrafish), or even the type of technique used to validate stem cell fate (lineage labeling) or stem cell potential (transplantation, clonogenicity, lineage tracing). Taking into account that cellular plasticity will enable cells that *a priori* do not exhibit stem cell properties to acquire stem cell potential if needed (self‐renew and differentiate), we therefore propose a more reconciled concept, whereby liver cells possess an extreme plasticity that allows the acquisition of different states (differentiation‐stemness) depending on the environment and tissue demand (Fig. 1A).

## Isolation of Liver Cells With Clonogenic and Multilineage Potential

As mentioned, different experimental approaches have been used to identify stem cells or cells with stem cell potential: from lineage tracing to transplantation or colony formation (see Fig. 1).

Using antibodies and/or flow cytometry‐based cell separation methods, several groups have actually managed to isolate cell populations from the adult liver.^31^ Here, we will focus on the use of cellular markers that identify liver cells with clonogenic and multilineage potential.^9^, ^32^ EpCAM,^16^ Lgr5,^22^ CD133,^33^ MIC1‐1C3,^33^ Foxl1,^19^ OPN,^12^ Sox9,^20^ and CD24^34^ markers or antibodies, or a combination of them, have been mostly used to enrich for cells that, upon culture and/or transplantation, exhibit clonogenic and multilineage competency. Also, activities (functionality of the cell) that are enhanced in stem/progenitor cells can be used to isolate putative cells with stem cell potential, for instance, aldehyde dehydrogenase activity (Fig. 1B).^35^ Unfortunately, the aforementioned markers are usually expressed on regular biliary epithelial cells, which complicates their isolation. Similarly, expression of markers has been shown in a subpopulation of rat progenitors but is not found in the mouse counterpart. Conversely, OPN and MIC1‐1C3 are regarded as equivalent progenitor markers, at least in mice. Also, some markers appear only upon liver injury (like Lgr5 or Foxl1), while they are not present under homeostasis conditions. Together with the fact that stem/progenitor cell populations represent a spectrum of differentiation states, this makes the development of a unified isolation strategy difficult.

As a general view, assuming that liver stem cells are individual entities carrying specific markers is rather outdated. Perhaps our thinking on how liver stem cells work (and probably the parameters used to define stem cells) has been mistaken. For instance, it is becoming clear that the quiescent state is far from being a protected state, as used to be thought.^36^, ^37^, ^38^ Liver repair is also achieved by expansion of many cells, with plasticity of the stem cells and mature cells and dedifferentiation emerging as common themes (Fig. 1A). For instance, by switching on cellular and metabolic plasticity upon response to injury, the rates and types of cell production have to be rapidly adjusted to meet the tissue's cellular and metabolic requirements.^38^, ^39^ Could it be that the markers cited earlier are involved in these matters? In the future, it would be rewarding to examine whether such critical events may be correlated to the presence of the particular aforementioned markers. In this concise review, we focus on Lgr5 and EpCAM as markers that could potentially offer identification of such plasticity.

## EpCAM as a Marker of Liver Cells During Homeostasis

**Figure 2:**
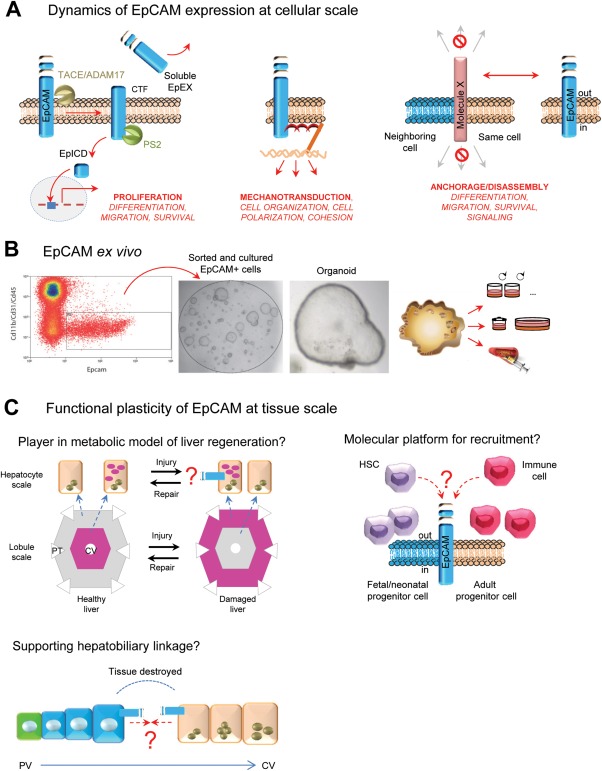
EpCAM as a marker of liver cells during homeostasis. (A) Dynamics of EpCAM expression at the cellular level. The pleiotropic functions of EpCAM can be allocated to the full‐length protein as well as to EpCAM‐derived fragments generated upon intramembrane proteolysis.^40^ Some functions are illustrated. (B) *Ex vivo*, sorted and cultured EpCAM^+^ cells are able to form organoids, with a high degree of plasticity.^22^, ^47^ (C) Functional plasticity of EpCAM at the tissue scale is illustrated: EpCAM could be a player in a metabolic model of liver regeneration or as a molecular platform for cell recruitment. EpCAM expression on peribiliary hepatocytes (namely, canal of Hering‐associated hepatocytes found at the hepatocyte‐biliary interface) could allow an efficient hepatobiliary linkage to drain bile. Abbreviations: EpEX, EpCAM large N‐terminal extracellular domain; EpICD, EpCAM short C‐terminal fragment; ADAM, a disintegrin and metalloprotease; CTF, C‐terminal fragment; CV, central vein; PS2, presenilin‐2; PV, portal vein; TACE, tumor necrosis factor‐alpha‐converting enzyme.

EpCAM is a transmembrane glycoprotein that is frequently expressed in cancer.^40^ EpCAM is composed of a large N‐terminal extracellular domain (called EpEX) linked to a short C‐terminal fragment (named EpICD) by a single‐transmembrane domain (see Fig. 2A). Recently, EpCAM was recognized as a marker for pluripotent stem cells in humans and mice and for tissue stem cells (reviewed in Dollé et al.^40^). EpCAM can interact with proteins like E‐cadherin or claudins to modulate cell‐cell contact, regulate the activity of signaling pathways, or sequester molecules or receptors to prevent their biological effects (see Fig. 2A).^40^ EpCAM is a potent player in the maintenance of the polarized tissue and has been described to modulate the organization of the actin cytoskeleton^41^ and actomyosin contractility.^42^ While only its proliferative effect has formally been demonstrated,^43^ it is tempting to propose that EpCAM regulates the actomyosin network for functional purposes.

Remarkably, EpCAM expression is not restricted to epithelial precursors but is also present in undifferentiated stem cells that are not yet assigned to a specific cell fate. During morphogenesis of pancreatic islets, EpCAM has been described as a morphoregulatory molecule^44^ whereby it is highly expressed in fetal endocrine pancreas, while the adult endocrine tissue exhibits low levels of expression. This developmentally regulated EpCAM expression has also been illustrated in other organs, such as kidney, lung, skin, and thymus (reviewed in Dollé et al.^40^). During liver development and homeostasis, EpCAM also demonstrates dynamic expression as it can be detected in immature cells, which gradually lose it along with their maturation into hepatocytes.^16^, ^45^, ^46^ So far, EpCAM is one of the most representative and successful markers used in isolating liver stem cells (Fig. 1B). Notably, long‐term culture of genome‐stable EpCAM^+^ bipotent stem cells from adult human liver has been developed (Fig. 2B).^47^

Still many questions regarding the role of EpCAM in liver regeneration remain unanswered. While *in vitro* these cells show bipotential competency, *in vivo* the reason for reexpression of EpCAM remains speculative (see Fig. 2C). Data by Yoon et al. clearly indicate the existence of a hierarchically structured regeneration of the liver based on differentiation processes that require the reexpression of EpCAM.^48^ Recently, a possible perspective on the role of EpCAM in the maturation of human hepatocyte buds has been elegantly shown. Briefly, the authors demonstrated that hepatocyte buds derived from progenitor cells (i.e., glutamine synthetase‐positive/EpCAM^+^ cells) and repopulate regions of extinct parenchyma in human cirrhosis by following a maturation process that involves dynamic expression of EpCAM and glutamine synthetase,^49^ allowing us to think that EpCAM might be required for stem cell maturation. Furthermore, the associated microvasculature develops in concert with the maturation of buds, resulting in a loss of CD34 expression in the bud center with the development of well‐defined sinusoids, while the periphery sustains a CD34 positivity matching the dynamics of EpCAM expression.^49^ This potentially exhibits EpCAM as a molecular platform permitting endothelial cell (CD34^+^) recruitment to ensure correct liver cell differentiation. A similar scenario has been speculated for explaining the hematopoietic cell migration from the fetal liver to the adult bone marrow (Fig. 2C).^40^ In response to injury, the plasticity of the hepatobiliary system has been recently unveiled.^24^ Interestingly, in this study EpCAM^+^ cell density matched the distance traveled by the emerging biliary branches. Consequently, one could consider that EpCAM has a role in this structural flexibility or possibly in the directionality of the biliary branches.

Several reports have demonstrated that immediately after an injury drastic changes in metabolism occur in the liver before the repair machineries are launched.^50^, ^51^, ^52^ Disturbance of the metabolic zonation upon injury led to the hypothesis of whether sensing of this metabolic insufficiency may in fact be the initiating trigger for the regenerative response. It is then tempting to speculate that *de novo* EpCAM expression on adult hepatocytes in the lobular parenchyma in response to injury could be an adaptive response to compensate for the hepatic insufficiency by creating a different metabolic zonation (see Fig. 2C). In this viewpoint, cellular plasticity of EpCAM, at cellular or tissue scale, is important because at one site (e.g., stem/progenitors) EpCAM might be dedicated for proliferation and at another site (e.g., hepatocytes) it can be required for response to hepatic insufficiency.

Overall, recent studies highlight the importance of the epithelial diversity that surrounds the bile ducts, which probably could partially explain the extraordinary plasticity of the biliary tree. Intriguingly enough, by (re)expressing EpCAM at cellular or tissue scale or by exposing a different integrity of the full‐length EpCAM molecule, the liver cells are champions of cellular plasticity. Whether EpCAM has a role in liver tissue plasticity remains an open question.

## Lgr5 as a Marker of Liver Cells Following Damage

**Figure 3:**
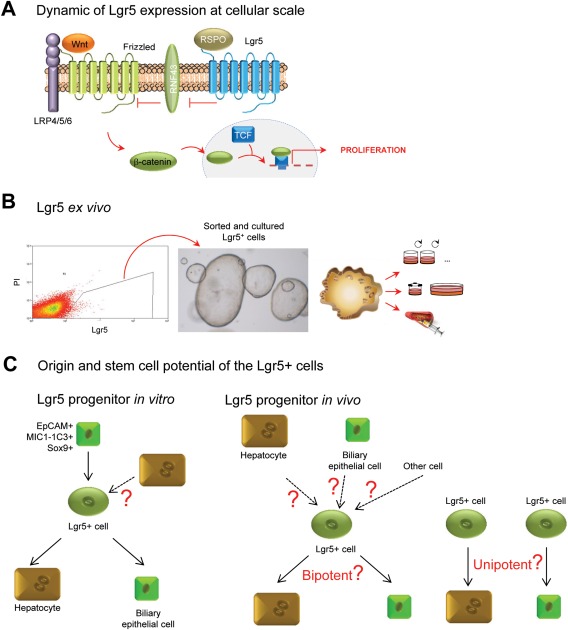
Lgr5 as a marker of liver cells following damage. (A) Dynamics of Lgr5 expression at the cellular level. (B) *Ex vivo*, sorted and cultured Lgr5^+^ cells are able to form organoids, with a high degree of plasticity.^22^, ^47^ (C) *In vitro*, Lgr5 cells derive from biliary epithelial cells.^47^, ^60^ Because medium to grow hepatocytes in culture has not been established yet, the origin of Lgr5^+^ cells from hepatocytes cannot be addressed. *In vitro*, Lgr5 cells are bipotential, generating the two epithelial liver lineages, hepatocytes and biliary epithelial cells.^22^, ^47^ The cell of origin of Lgr5^+^ cells *in vivo* is still unknown. *In vivo*, Lgr5 cells trace into hepatocytes and biliary epithelial cells.^22^ Whether *in vivo* these Lgr5^+^ cells can generate both lineages or there are Lgr5 committed progenitors to each lineage is still unknown. Abbreviations: RSPO, R‐spondin; PI, propidium iodide; TCF, T‐cell factor.

LGR5 is a G protein‐coupled receptor with a seven‐transmembrane domain. Together with its paralogue, LGR4, it is crucial for maintaining proliferating progenitors and stem cells in the intestine.^53^ Biochemical analyses have identified the LGRs as receptors for R‐spondins.^54^ Following association with R‐spondins, LGR4/5/6 strongly promote the activity of Wnt‐Frizzled mediated signaling (Fig. 3A). In fact, R‐spondin‐LGR binding results in removal of the E3 ubiquitin ligase RNF43, thus preventing the degradation of Frizzled, which results in a more robust and prolonged Wnt signal emanating from a “stabilized” Wnt/frizzled complex (reviewed in Koo and Clevers^54^). Lineage tracing studies have confirmed that Lgr5^+^ cells are fast‐dividing, long‐lived adult stem cells in the hair follicles, the antropyloric stomach, and the gut (reviewed in Koo and Clevers^54^). Also, the mammary epithelium, the developing kidney, the ovarian epithelium, and supporting cells in the inner ear possess LGR5^+^ cells (reviewed in Koo and Clevers^54^).

In the liver, Wnt signaling is active in perivenous hepatocytes^55^ and has been shown to induce metabolic zonation of the liver lobule.^56^ Upon damage, either by hepatectomy,^57^ oval cell response,^58^ or central vein damage,^22^ Wnt signaling is highly activated (the role of Wnt and its effector, beta‐catenin, is elegantly reviewed in Nejak‐Bowen and Monga^55^ and is not the focus of this review). While classical canonical Wnt target genes, such as Axin 2, are detected in homeostasis in centrilobular hepatocytes, reporter mice have failed to show expression of Lgr5 under normal physiological conditions,^22^ although RNA analysis indicates basal expression of Lgr5 in this area.^59^ However, following liver damage, *Lgr5*, similarly to *Foxl1*,^19^ marks a population of cells that proliferates and, as shown by lineage tracing, upon damage caused by carbon tetrachloride, 3,5‐diethoxycarbonyl‐1,4‐dihydrocollidine, or methionine and choline‐deficient diet supplemented with ethionine, differentiate into hepatocytes and/or ductal cells.^22^ *In vitro* (Fig. 3B), these damage‐induced Lgr5^+^ cells exhibit stem cell potential; they can be expanded from single cells (clonogenic) into self‐sustaining liver organoids, while at the same time being able to differentiate toward cholangiocytes and hepatocytes (bipotentiality) *in vitro* and *in vivo*, after transplantation in the FAH^‐/‐^ mouse model.^22^ Unfortunately, Lgr5^+^ cells have not been transplanted in other liver disease models, such as following partial hepatectomy or injury from liver toxins. These models have proven very useful for the analysis of bipotentiality and stem cell behavior of neonatal and adult EpCAM^+^ cells derived from human donors.^16^, ^17^ Future studies are expected to answer this question.

Still, many questions regarding the role of Lgr5 in liver regeneration remain unresolved. While *in vitro* these cells show bipotential competency, their behavior *in vivo* is still unknown (Fig. 3C). In fact, a drawback of the lineage tracing experiments using an Lgr5Cre driver is that these experiments were not performed at the clonal level (as discussed above). Therefore, whether *in vivo* these Lgr5^+^ cells that appear after damage are bipotential or indeed there are two types of Lgr5 progenitors for the hepatocyte and ductal lineages (Fig. 3C) remains unresolved. Also, because this marker only appears after damage, the cell of origin from which these Lgr5^+^ cells arise *in vivo* is still unknown. Of note, *in vitro* mouse ductal MIC1‐1C3^+^ cells^60^ or human EpCAM^+^ liver cells generate liver organoids that express LGR5.^47^ Whether *in vivo* EpCAM^+^ cells are the cells of origin of *Lgr5* damage‐induced cells is unknown.

Because of the essential role of Lgr5 in enhancing Wnt signaling, it is tempting to hypothesize that LGR5 could be sensing higher levels of Wnt upon damage, which in turn could be inducing an active proliferative response on those specific cells to repair the tissue and reinstate homeostasis. It is worth mentioning that the dynamics of Lgr5 expression following injury indicate that LGR5 should be expressed early after the onset of damage and should be switched off again once the tissue is regenerated.^22^ Thus, it is plausible to speculate that Lgr5 could be acting as a switch between on and off states that instructs the cells whether to proliferate or not depending on the levels of Wnt in the environment. If that is the case, then Lgr5 would be marking cells that exhibit high plasticity and can move back and forth between different stem and differentiation states. If so, it is feasible to hypothesize that perturbations in the system could break the fine line between proliferation and differentiation and result in disastrous consequences such as tissue hyperproliferation (cancer) or degeneration (cirrhosis). With respect to that option, it has been recently shown that murine liver cancer cells have a similar expression pattern to Lgr5 liver progenitors induced after damage,^61^ suggesting that deregulation of a Wnt‐driven regenerative response could be a factor contributing to liver cancer. Of note, hepatocellular carcinomas harbor mutations in beta‐catenin or other Wnt pathway components, which could be reflecting a mechanism of the tissue to activate proliferation by enhancing Wnt signaling.

Overall, we are just beginning to understand the role of Lgr5 in stem cell maintenance and repair. In the liver, future studies will be required to identify the cells from which Lgr5^+^ damaged cells arise and the role of Lgr5 during regeneration. Whether in the liver Lgr5 is implicated in tissue plasticity remains an open question.

## Conclusions

It is well established that high proliferative tissues such as the gut and the skin have evolved mechanisms to prevent tissue degeneration in the event of damage to their bona fide stem cell compartments. Thus, ablation of the stem cell pool results in activation of “reserve” populations or, also on the dedifferentiation of mature cell types toward a more stem cell state (potential/plasticity), to allow the repair of the tissue and reinstate homeostasis.^2^ Similarly, increasing evidence suggests that activation of a “stem cell state” in *a priori* non‐stem cell pools is not unique to the gut or the skin but occurs across many tissues. Thus, stomach^62^ and lung^8^ differentiated cells have also demonstrated the acquisition of stem cell properties (stem cell potential) upon damage to the tissue, in what are examples of cellular plasticity. Here, we have discussed the evidence on stem cell plasticity in the liver. The remarkable regenerative capacity of the liver under many different types of liver injury makes it a champion of cellular plasticity. Liver differentiated cells, potential resident stem cells, and even bone marrow stem cells can be dedifferentiated, activated, or recruited, respectively, to recover the damaged liver. This capacity of cells to acquire a stem cell state may highlight a more universal phenomenon. Whether this plasticity is relevant to disease states is yet to be determined, but recent evidence suggests that, at least in the intestine, dedifferentiation of non‐stem cells results in acquisition of a tumor‐initiating stem cell competency,^63^ thus highlighting the concept of bidirectional conversion and cellular plasticity as potentially relevant not only to tissue repair but also to tumorigenesis.

Author names in bold designate shared co‐first authorship.
